# Host plant resistance, foliar insecticide application and natural enemies play a role in the management of *Melanaphis sorghi* (Hemiptera: Aphididae) in grain sorghum

**DOI:** 10.3389/fpls.2022.1006225

**Published:** 2022-09-15

**Authors:** Osariyekemwen Uyi, Sriyanka Lahiri, Xinzhi Ni, David Buntin, Alana Jacobson, Francis P. F. Reay-Jones, Somashekhar Punnuri, Anders S. Huseth, Michael D. Toews

**Affiliations:** ^1^Department of Entomology, University of Georgia, Tifton, GA, United States; ^2^Department of Zoology and Entomology, Faculty of Natural and Agricultural Sciences, University of the Free State, Bloemfontein, South Africa; ^3^Department of Entomology, Gulf Coast Research and Education Center, University of Florida, Wimauma, FL, United States; ^4^Crop Genetics and Breeding Research Unit, USDA-ARS, Tifton, GA, United States; ^5^Department of Entomology, University of Georgia, Griffin, GA, United States; ^6^Department of Entomology and Plant Pathology, Auburn University, Auburn, AL, United States; ^7^Department of Plant and Environmental Sciences, Clemson University, Pee Dee Research and Education Center, Florence, SC, United States; ^8^College of Agriculture, Family Sciences and Technology, Fort Valley State University, Fort Valley, GA, United States; ^9^Department of Entomology and Plant Pathology, North Carolina State University, Raleigh, NC, United States

**Keywords:** invasive species, aphid, insect pest management, insecticide application, natural enemy

## Abstract

The invasive *Melanaphis sorghi* (Theobald; =*Melanaphis sacchari* Zehntner) is a serious pest of sorghum production in the southern USA. Demonstration of technologies that provide effective control is key to management of this pest. Here, we investigated the effect of host plant resistance (resistant cultivar: DKS37-07 and susceptible cultivar: DKS53-53) and a single foliar insecticide (flupyradifurone: Sivanto Prime) application on *M. sorghi* infestations and the role of natural enemy populations in grain sorghum production across five locations in four states in southeastern USA. Foliar insecticide application significantly suppressed *M. sorghi* infestations on both the resistant and susceptible sorghum cultivars across all locations. Planting the host plant resistant cultivar (DKS37-07) significantly reduced aphid infestation across all locations. Plant damage ratings did not vary widely, but there was generally a positive association between aphid counts and observed plant damage, suggesting that increasing aphid numbers resulted in corresponding increase in plant damage. Planting a host plant resistant cultivar and foliar insecticide application generally preserved grain yield. Both sorghum hybrids supported an array of different life stages of natural enemies (predators [lady beetle larvae and adults; hoverfly larvae and lacewing larvae] and parasitoids [a braconid and aphelinid]) for both the sprayed and non-sprayed treatments. We found a strong and significant positive relationship between the natural enemies and the *M. sorghi* infestation. Results suggest that planting a host plant resistant cultivar and the integration of natural enemies with insecticide control methods in the management of *M. sorghi* is central to the development of an effective pest management strategy against this invasive pest.

## Introduction

*Melanaphis sorghi* (Theobald) which was until recently known as *Melanaphis sacchari* Zehntner in previous literature (Nibouche et al., 2021), is an invasive multivoltine piercing and sucking pest of sugarcane, *Saccharum officinarum* (L.), and sorghum, *Sorghum bicolor* (L.) in Asia, Africa, Oceania, Central, South and North America (Sharma and Nwanze, 1997; Singh et al., 2004). *Melanaphis sorgi* was first detected on the Florida peninsula on the southeastern coast of the United States in 1977 and consequently only achieved a minor pest status in sugarcane production (Denmark, 1988; Mondor et al., 2006). Following the detection of a new haplotype of *M. sorghi* in Texas and Louisiana in 2013 (Harris-Shultz et al., 2017; Medina et al., 2017; Nibouche et al., 2018), the pest became a significant economic pest of sorghum (Bowling et al., 2016) and has since spread to 25 states in the southern United States, thus infesting all sorghum-production regions (Peterson et al., 2018; EDDMapS, 2020). As of the time, this aphid was originally misidentified as the sugarcane aphid, *Melanaphis sacchari*, until a recent study (Nibouche et al., 2021) based on morphological and molecular evidence revised its name to *M. sorghi*.

The rapid invasion success of *M. sorghi* may be partly due to its narrow host range (Armstrong et al., 2015;Haar et al., 2019; Harris-Shultz and Ni, 2021), capacity for dispersal (Bowling et al., 2016) and its potential to occupy a wide range of climatic conditions and ecosystems (Singh et al., 2004; Bowling et al., 2016; Souza and Davis, 2020), including disturbed ecosystems and agroecosystems where the preferred hosts are abundant (Singh et al., 2004; Bowling et al., 2016; Haar et al., 2019; Gordy et al., 2021; Harris-Shultz and Ni, 2021). *Melanaphis sorghi* can survive low temperatures (around 0°C) but does not undergo diapause, nor sexual reproduction in the United States (Bowling et al., 2016; Michaud et al., 2016). Populations overwinter on Johnson grass, *Sorghum halepense* (L.) and giant miscanthus, *Miscanthus sinensis* × *Miscanthus sacchariflorus* Greef & Deuter ex Hodkinson & Renvoize, in southern Alabama and Georgia (Haar et al., 2019; Harris-Shultz and Ni, 2021). Hot and dry weather conditions promote rapid population increases (Bowling et al., 2016) and hot weather events may further reduce the current doubling time of 4–13 days (Singh et al., 2004; Bayoumy et al., 2016; Brewer et al., 2017; Gordy et al., 2021). This may consequently lead to range expansion and rapid population increases that can limit grain yield in susceptible sorghum varieties and other economically important host crops.

In many states in southern United States, *M. sorghi* has become an important economic pest, causing significant yield loss in grain sorghum (Bowling et al., 2016; Peña-Martinez et al., 2016; Brewer et al., 2017; Szczepaniec, 2018; Lahiri et al., 2021), which translates into severe economic losses for farmers (Bowling et al., 2016). For example, the Louisiana sorghum industry suffered losses of approximately $7.7 million in 2013 due to *M. sorghi* (Kerns et al., 2015), while Georgia growers decreased the area planted to grain sorghum by nearly 60% from 2015 to 2017 due to severe infestations (Bostick et al., 2020). In Texas, annual economy-wide losses totaled $169.83 million in economic output including a direct loss of $78.57 million to farms and farm related industries (Zapata et al., 2018). At high densities, feeding by nymphs and adults of *M. sorghi* cause physiological stress in grain sorghum which causes chlorosis, leaf wilt and necrosis (Singh et al., 2004; Bowling et al., 2016). Feeding by *M. sorghi* also results in the production of copious amounts of honeydew, which promotes the growth of sooty mold on leaves, impeding photosynthesis of affected sorghum plants (Singh et al., 2004; Bowling et al., 2016). Further, sooty mold accumulation can clog grain sorghum harvest equipment (Singh et al., 2004; Bowling et al., 2016). Damage caused by *M. sorghi* decreases or stops grain sorghum growth, reducing crop yield by more than 50% and can kill susceptible grain sorghum plants (Bowling et al., 2016; Brewer et al., 2017; Gordy et al., 2019; Haar et al., 2019; Wilson et al., 2020; Lahiri et al., 2021).

Recent efforts focused on the development of economic thresholds (ET) as an integral tool to limit *M. sorghi* population growth provide decision support on insecticide timing within the framework of integrated pest management (IPM; Knutson et al., 2016; Gordy et al., 2019). Gordy et al. (2019) identified a range of 19–132 aphids per leaf as estimated ETs and suggested that a 40 aphid per leaf threshold across the range of cultivar., environmental, and market conditions in their study, however, this threshold needs revision for use on resistant sorghum cultivars. Previous studies demonstrate that knowledge of economic thresholds coupled with the use of aphid resistant sorghum varieties, insecticidal seed treatments, in-furrow or foliar insecticide sprays coupled with manipulation of planting date and nitrogen levels provide the basis for a comprehensive IPM program to manage *M. sorghi* in grain sorghum (Sharma et al., 2013; Armstrong et al., 2015; Etheridge et al., 2018; Szczepaniec, 2018; Haar et al., 2019; Paudyal et al., 2019; Seiter et al., 2019; Wilson et al., 2020; Lahiri et al., 2021; Lytle and Huseth, 2021; Pekarcik and Jacobson, 2021). Further evaluation of factors including insecticide application and host plant resistance influencing *M. sorghi* infestations and resulting yield losses is necessary to improve IPM strategies. The use of resistant cultivars provides a baseline of protection against *M. sorghi* by suppressing population growth rates, limiting injury and improving grain yield, however, the performance of these varieties is geographically variable (Lahiri et al., 2021; Pekarcik and Jacobson, 2021). Application of foliar insecticides such as flupyradifurone (Sivanto Prime, Bayer CropScience, Research Triangle Park, NC, United States) clearly suppress *M. sorghi* populations (Lahiri et al., 2021; Pekarcik and Jacobson, 2021), but the efficacy of foliar application may vary by weather conditions or geographic locations (Lahiri et al., 2021). Hence, continuous studies on the influence of host plant resistance and foliar insecticide application across locations are needed. These studies could potentially show how to improve the efficacy of natural enemies of *M. sorghi* in the management of this pest.

Knowledge of the non-target impacts of insecticides used for *M. sorghi* can enable growers to make informed decisions about insecticide selection that decrease aphid infestation while preserving the abundance and activities of natural enemies. Several studies have recorded a multiplicity of predators and parasitoids in sorghum production systems in southern United States suggesting that natural enemies may play a role in suppressing *M. sorghi* population, especially at low densities (Singh et al., 2004; Hewlett et al., 2019; Maxson et al., 2019; Lytle and Huseth, 2021). Despite the fact that previous studies documented more than 47 arthropod species feeding on *M. sorghi* (Singh et al., 2004; Lytle and Huseth, 2021), not much is known about the role of natural enemies on *M. sorghi* in grain sorghum systems in the southeastern USA due to prolific aphid reproduction rate, and low natural enemies in the sorghum field at the initial aphid infestation (but see Lytle and Huseth, 2021). Understanding the role of insect natural enemies in grain sorghum systems that incorporate foliar insecticide sprays and host plant resistance across multiple locations in the United States is crucial to refining our IPM strategies in managing this pest. The objective of this study was to investigate the efficacy of combining host plant resistance and foliar insecticidal application using two commercial grain sorghum cultivars (susceptible cultivar: DKS53-53 and resistant cultivar: DKS37-07) across five locations, in four southeastern states in the United States. A second objective of this study was to determine if predators and parasitoids play some role in managing *M. sorghi* populations in grain sorghum systems that combine host plant resistance and foliar insecticide application within the context of IPM.

## Materials and methods

### Study locations and agronomic practices

Between April and August 2018, large plot field experiments utilizing grain sorghum were conducted at Tift Co., Georgia (31.5120° N, −83.6434° W), Pike Co., Georgia (33.1779° N, −84.4090° W), Moore Co., NC (35.1840° N, −79.6779° W), Barbour Co., Alabama (32.4224° N, −85.8907° W), and Darlington Co., South Carolina (34.3650° N, −80.0088° W). At each trial location, cooperators followed state Cooperative Extension recommended agronomic practices to achieve a 5,406 kg/ha yield goal. After spreading the recommended amounts of dry fertilizer, the fertilizer was incorporated using a field cultivator and then seedbed preparation was accomplished with a one-pass ripper bedder with the subsoil shank set to a depth of 50.8 cm for breaking the hardpan under the rows. A total of eight adjacent plots (11 m by 30.5 m per plot) were delineated and planted using a vacuum planter in early to mid-May. Plots were laid out on 0.9 m row centers at a planting density of 247,105 seeds per ha and a depth of 3.8-cm. A total of four plots received an *M. sorghi* susceptible grain sorghum cultivar., DKS53-53 (DeKalb®, Monsanto Company, St. Louis, MO, United States), while the remaining four plots received an *M. sorghi* resistant cultivar., DKS37-07. Sorghum seeds were treated with fluxofenin (Concep III, Syngenta Crop Protection, Greensboro, NC) to permit application of S-metolachlor (Dual Magnum, Syngenta Crop Protection) at 1.17 L/ha behind the planter for enhanced weed control. One month after planting, all plots received atrazine (AAtrex 4 l, Syngenta Crop Protection) at 2.63 L/ha to provide additional weed suppression.

### Insecticide treatment

Mean number of *M. sorghi* across all plots were summarized weekly. When the aphid population across the entire trial reached 50 aphids per bottom leaf, a rescue insecticide treatment was initiated in two plots planted with resistant cultivar and two plots planted with susceptible cultivar. Those plots received a one-time application of flupyradifurone (Sivanto Prime, Bayer CropScience, Rhein, Germany) at 0.36 L/ha, administered using a self-propelled sprayer equipped with hollow cone nozzles (model TXVS-8, TeeJet Technologies, Spraying Systems Co., Glendale Heights, IL). Applications were delivered in a spray volume of 93.5 L/ha.

### Insect sampling and plant health assessment

*Melanaphis sorghi* abundance and plant condition were assessed weekly. Weekly assessments started 4 weeks after planting and continued until the grain reached the hard dough stage in mid-August of 2018 at all locations for up to 8 weeks. *Melanaphis sorghi* (regardless of age) and natural enemies were sampled from a single lower and upper leaf from six randomly selected plants per plot. To avoid edge effects, plants were sampled from the center two rows. All nymphs, alate, and apterous adult aphids were aggregated into a single count per leaf. Exact aphid numbers were counted when the density was below 50, and when densities were above 50, the number of aphids were estimated. We recorded the presence and number of beneficial insects spanning 11 taxa that are known predators of *M. sorghi* (Singh et al., 2004; Bowling et al., 2016; Lytle and Huseth, 2021). We identified parasitoid wasps [*Lysiphlebus testaceipes* (Cresson; Hymenoptera: Braconidae) and *Aphelinus* sp. (Hymenoptera: Aphelinidae)] by characterizing aphid mummies.

To simultaneously account for aphid abundance and duration of infestations, aphid counts were converted to cumulative insect days (CID) on a per plot basis following the methods of Ruppel (1983). Briefly, aphid days were calculated for each sampling interval as the mean density of two consecutive sample dates multiplied by the length of the interval between the dates in days. These values accumulated over the entire sampling period in each year, providing a cumulative estimate of aphid infestation intensity for each plot. On a per plot basis, plant condition (or aphid damage) of six randomly selected plants was characterized on a scale of 1–9 following the methods of Sharma et al. (2013). Briefly, this scale provides a standardized method to describe *M. sorghi* infestations based on the number of leaves showing damage symptoms and honeydew/sooty mold accumulation.

### Harvest

When the grain dried in the field to a moisture content of 15% or less, the center two rows from each plot were harvested using a self-propelled combine. Depending on location, harvest generally commenced in late August to early October. Grain yield in each location and moisture content were measured on the combine. For comparison purposes, all plots were adjusted to a common 14% moisture content and extrapolated to kg of grain per ha.

### Data analysis

At each location, experiments were organized in a factorial arrangement of treatments nested in a randomized complete block design. Treatments were cultivar (DKS53-53 vs. DKS37-07) and insecticide application (sprayed vs. unsprayed). The experimental unit receiving treatments (cultivar and insecticide treatment) was an individual grain sorghum plot measuring 11 m by 30.5 m. Responses averaged across individual plots included aphid counts per leaf, cumulative insect days (CID)—an index of crop protection which simultaneously account for the severity and duration of aphid infestation as described by Ruppel (1983), counts of natural enemies on each of 6 random plants per plot, and plant damage estimates. Plant damage ratings at Barbour Co. were not recorded. Following square root transformation of CID and damage rating data, the effects of sorghum hybrids and foliar insecticide application on CID and damage rating was analyzed using a Generalized Linear Model (GLZ; assuming normal distribution with an identity link function). When the overall results were significant in the GLZ analysis, the difference among the treatments was compared using the sequential Bonferroni test. The effect of sorghum cultivar (DKS37-07 vs. DKS53-53) and insecticide application on sorghum yield was evaluated using univariate General Linear Model analysis of variance (GLM ANOVA). When the overall results were significant in a two-way analysis, the differences among the treatments were compared using the Tukey’s Honest Significant Difference (HSD) test. We pooled yield data across all four locations and analyzed the overall effect of cultivar and insecticide treatment on grain sorghum yield, using GLM ANOVA. Regression analysis between number of *M. sorghi* and plant damage rating was only performed for the sprayed and non-sprayed susceptible sorghum cultivar (DKS53-53), because *M. sorghi* numbers and damage rating on the resistant hybrid was very low. Natural enemy counts were mostly zeros across locations, hence we pooled the data across all study locations and represented it as pie charts according to sorghum cultivar and insecticide application. Irrespective of sorghum cultivar and insecticide application, we combined all-natural enemy data (extremely very low) and performed regression analysis on the relationship between the number of *M. sorghi* and natural enemy’s abundance. Except for the regression analyses that were performed using Microsoft Excel and GENSTAT 9.0 (VSN International, Hemel Hempstead, United Kingdom), all other analyses were performed using IBM SPSS Statistical software version 20.0 (SPSS, Chicago, IL, United States).

## Results

### Cumulative insect days

**Figure 1:**
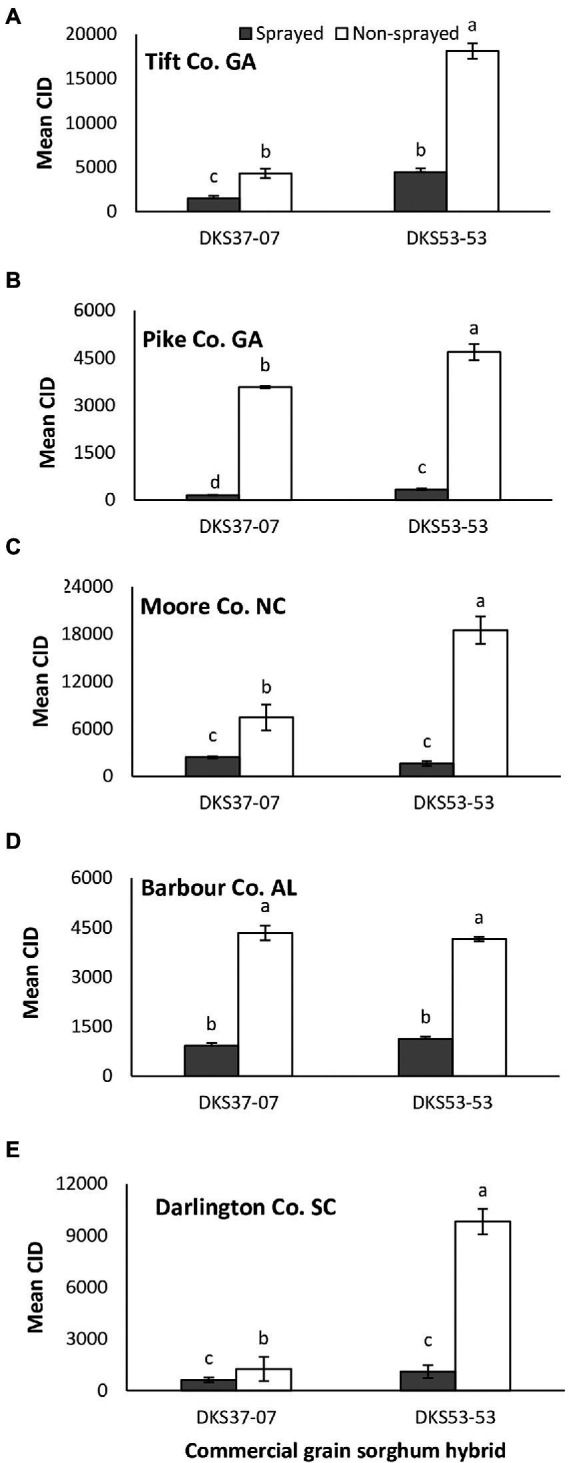
Mean (±SE) cumulative insect days (CID) in flupyradifurone sprayed and non-sprayed grain sorghum cultivars planted at Tift and Pike Co. GA, Moore Co. NC, Barbour Co. AL and Darlington Co., SC in 2018. Means capped with different letters are significantly different (sequential Bonferroni test, *p* < 0.05) among all four treatments. Note that y-axis scales are different on each figure.

*Melanaphis sorghi* infestation as indicated by CID was significantly influenced by sorghum cultivar and foliar insecticide application across study locations. Specifically, there were differences at Tift Co., GA (sorghum cultivar: *χ*^2^ = 228.35, *p* = 0.001; insecticide application: *χ*^2^ = 220.86, *p* = 0.001; interaction: *χ*^2^ = 95.72, *p* = 0.001), Pike Co., GA (sorghum cultivar: *χ*^2^ = 12.59, *p* = 0.001; insecticide application: *χ*^2^ = 470.31, *p* = 0.001; interaction: *χ*^2^ = 6.82, *p* = 0.009) and Moore Co., NC (sorghum hybrid: *χ*^2^ = 28.75, *p* = 0.001; insecticide application: *χ*^2^ = 131.22, *p* < 0.001; interaction: *χ*^2^ = 38.44, *p* = 0.001; Figures 1A–C). In Tift Co., *M. sorghi* infestation was 2-fold and 3-fold higher for non-sprayed (relative to sprayed) resistant cultivar (DKS37-07) and susceptible cultivar (DKS53-53), respectively (Figure 1A). In Pike Co., CID values was 20- and 13-fold higher for non-sprayed (relative to sprayed) resistant cultivar (DKS37-07) and susceptible cultivar (DKS53-53), respectively (Figure 1B). At Moore Co., CID was 2- and 10-fold higher for non-sprayed resistant cultivar (DKS37-07) and susceptible cultivar (DKS53-53), respectively (Figure 1C). Irrespective of foliar insecticide application, the resistant cultivar (DKS37-07) significantly reduced *M. sorghi* infestation compared to the susceptible cultivar (DK53-53) in Tift, Pike, and Moore Counties (Figures 1A–C). Cumulative insect days was not significantly influenced by sorghum cultivar but differed according to foliar insecticide application in Barbour Co., AL (sorghum cultivar: *χ*^2^ = 0.002, *p* = 0.965; insecticide application: *χ*^2^ = 169.62, *p* = 0.001; interaction: *χ*^2^ = 0.626, *p* = 0.429; Figure 1D); where CID was higher on non-sprayed plots for both sorghum cultivars. Finally, CID was significantly higher in non-sprayed sorghum plots and on susceptible sorghum cultivar in Darlington Co., SC (sorghum cultivar: *χ*^2^ = 57.32, *p* = 0.001; insecticide application: *χ*^2^ = 142.86, *p* = 0.001; interaction: *χ*^2^ = 12.042, *p* = 0.001; Figure 1E).

### Plant damage rating

**Figure 2:**
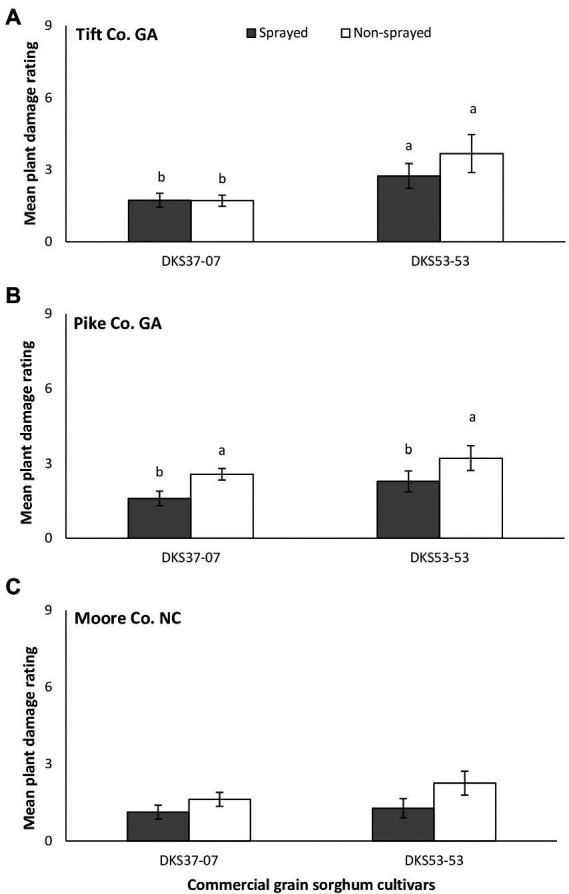
Mean (±SE) plant damage rating in flupyradifurone sprayed and non-sprayed grain sorghum cultivars planted at Tift and Pike Co. GA and Moore Co. NC. Means capped with different letters are significantly different (sequential Bonferroni test, *p* < 0.05) among all four treatments.

In Tift Co., a significant difference in plant damage rating was detected between sorghum hybrids and there was always more damage to the susceptible cultivar compared to the resistant cultivar (*χ*^2^ = 9.05, *p* = 0.003), but damage did not differ as a function of foliar insecticide application (*χ*^2^ = 0.853, *p* = 0.356; Figure 2A). There was no significant interaction between sorghum cultivar and insecticide application (*χ*^2^ = 0.924, *p* = 0.336). Plant damage rating did not vary as a function of sorghum cultivar (*χ*^2^ = 1.882, *p* = 0.170) but was greater on the non-sprayed (compare to sprayed) susceptible and resistant sorghum cultivars (*χ*^2^ = 3.850, *p* = 0.05) in Pike Co. (Figure 2B). There was no significant interaction between sorghum cultivar and insecticide application (*χ*^2^ = 0.002, *p* = 0.965). In Moore Co., there was no significant effect of sorghum cultivar (*χ*^2^ = 0.384, *p* = 0.536), foliar insecticide application (*χ*^2^ = 2.676, *p* = 0.102) or interaction between sorghum cultivar and insecticide application (*χ*^2^ = 0.763, *p* = 0.382) on plant damage rating (Figure 2C).

**Figure 3:**
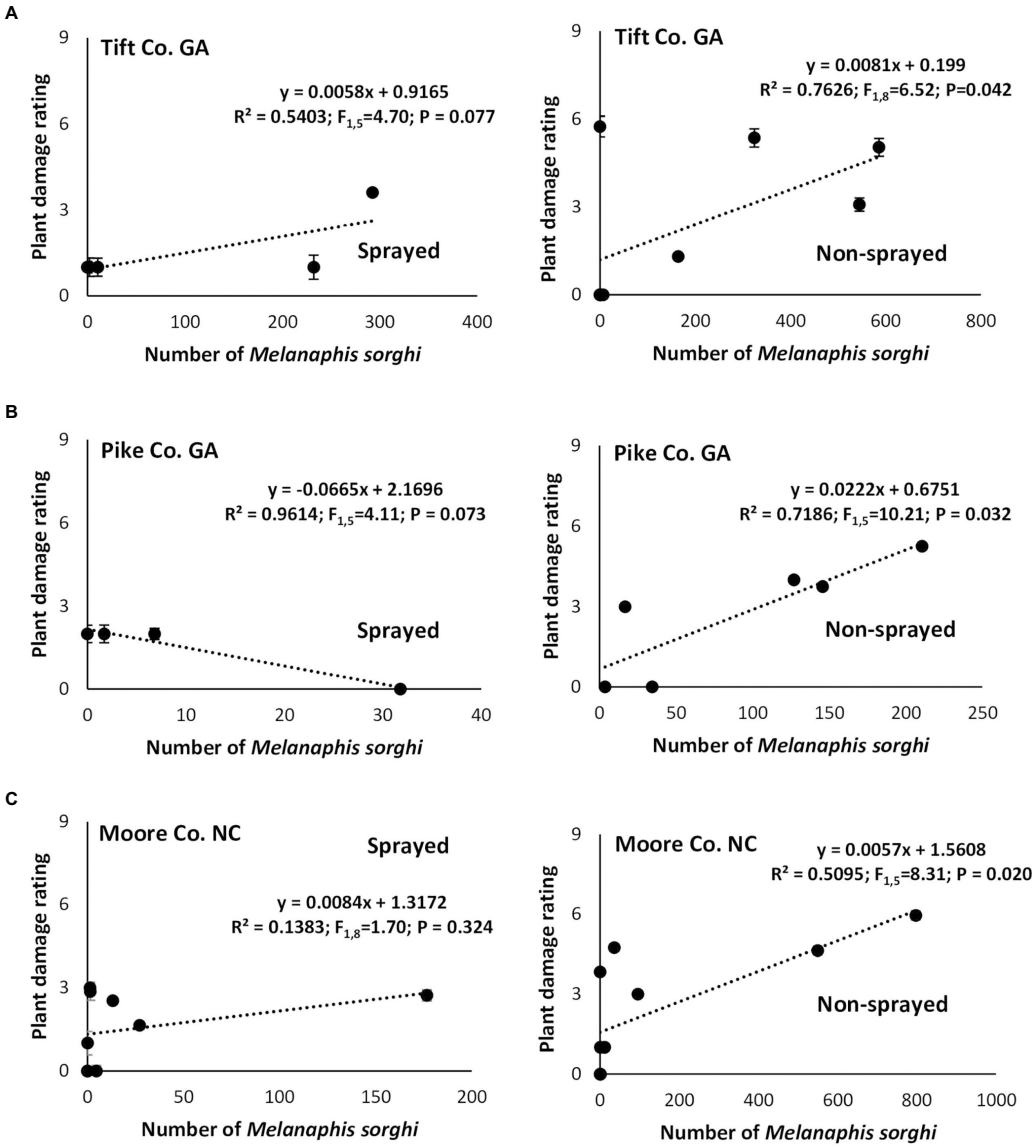
Relationship between mean weekly *Melanaphis sorghi* counts and corresponding plant damage rating in flupyradifurone sprayed and non-sprayed susceptible sorghum cultivar (DKS53-53) at Tift Co. **(A)**, Pike Co. GA **(B)**, and Moore Co. NC **(C)** in 2018.

In Tift Co., irrespective of foliar insecticide application, linear regression analysis showed positive relationships between the number of *M. sorghi* and the resulting plant damage ratings, however, only the non-sprayed treatment showed a significant association (Figure 3A). In Pike Co., a non-significant negative relationship between the number of *M. sorghi* and plant damage rating was evident on the sprayed susceptible sorghum cultivar while the non-sprayed susceptible sorghum cultivar had a significant strong linear relationship between the number of *M. sorghi* and plant damage rating (Figure 3B). Finally, in Moore Co., a non-significant weak relationship between the number of *M. sorghi* and plant damage rating was evident in the sprayed susceptible sorghum cultivar while the non-sprayed susceptible sorghum cultivar had a significant positive linear relationship between the number of *M. sorghi* and plant damage rating (Figure 3C).

### Sorghum yield

**Figure 4:**
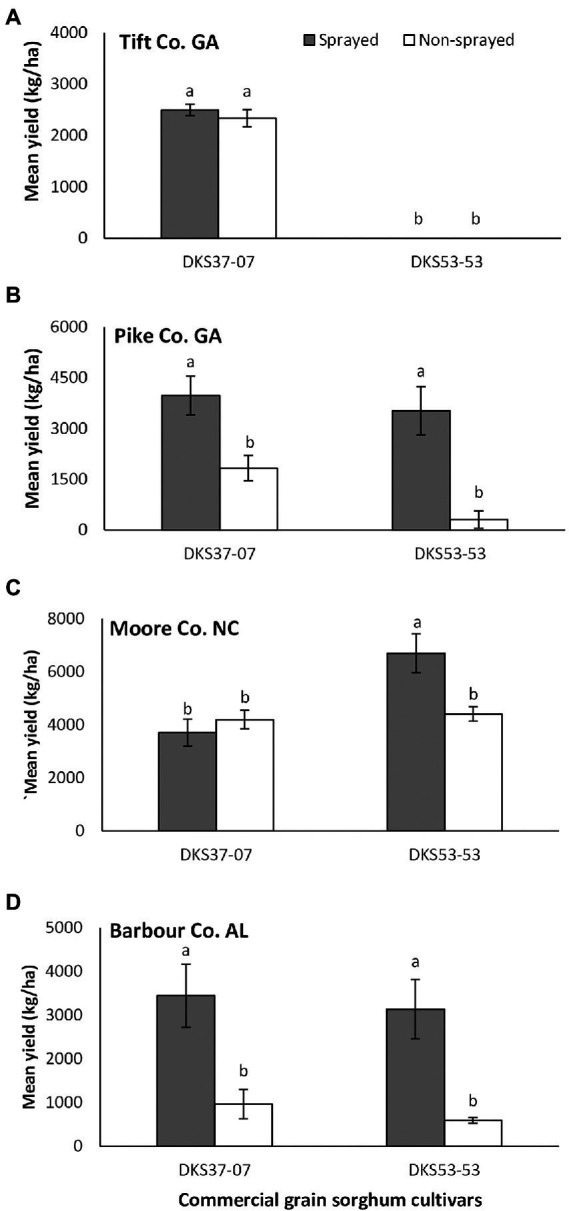
Mean (±SE) grain yield (kg/ha) as a function of sorghum cultivar and flupyradifurone foliar application at Tift Co., Pike Co. GA, Moore Co. NC and Barbour Co. AL. Means capped with different letters are significantly different (*p* < 0.05; Turkey’s test) among all four treatments. Note that y-axis scales are different on each figure.

Overall, grain sorghum yield was not significantly influenced by sorghum cultivar and foliar insecticide application when data from all four locations where pooled (cultivar: *F*_1,61_ = 1.31; *p* = 0.257; insecticide application: *F*_1,61_ = 3.38; *p* = 0.071; interaction: *F*_1,61_ = 0.68; *p* = 0.415), however, differences were detected in individual locations. In Tift Co., the resistant cultivar out yielded the susceptible cultivar (*F*_1,20_ = 581.60; *p* = 0.001), but did not differ as a function of foliar insecticide application (*F*_1,20_ = 0.616, *p* = 0.442; Figure 4A). There was no significant interaction between sorghum cultivar and insecticide application (*F*_1,20_ = 0.624, *p* = 0.446). Sorghum yield did not vary as a function of sorghum cultivar (*F*_1,4_ = 3.73, *p* = 0.126) but was significantly influenced by foliar insecticide application (*F*_1,4_ = 27.48, *p* = 0.006); with the sprayed treatment demonstrating evidently higher yield compared to non-sprayed for both sorghum cultivars in Pike Co. (Figure 4B). There was no significant interaction between sorghum hybrid and insecticide application nor the interaction of both factors (*F*_1,4_ = 1.12, *p* = 0.352). In Moore Co., there was a significant effect of sorghum cultivar (*F*_1,20_ = 10.37, *p* = 0.004) on sorghum yield with the susceptible hybrid producing higher yield compared to the resistant hybrid (Figure 4C). There was no effect of foliar insecticide application (*F*_1,20_ = 3.27, *p* = 0.088) on sorghum yield, but there was a significant interaction between sorghum cultivar and insecticide application (*F*_1,20_ = 7.76, *p* = 0.011). In Barbour Co., sorghum productivity did not vary as a function of sorghum cultivar (*F*_1,4_ = 0.61, *p* = 0.479) but was significantly influenced by foliar insecticide application (*F*_1,4_ = 33.19, *p* = 0.004); with the sprayed treatment demonstrating evidently higher yield compared to non-sprayed for both sorghum cultivars (Figure 4D). There was no significant interaction between sorghum cultivar and insecticide application (*F*_1,4_ = 1.04, *p* = 0.313).

### Natural enemy abundance and association

**Figure 5:**
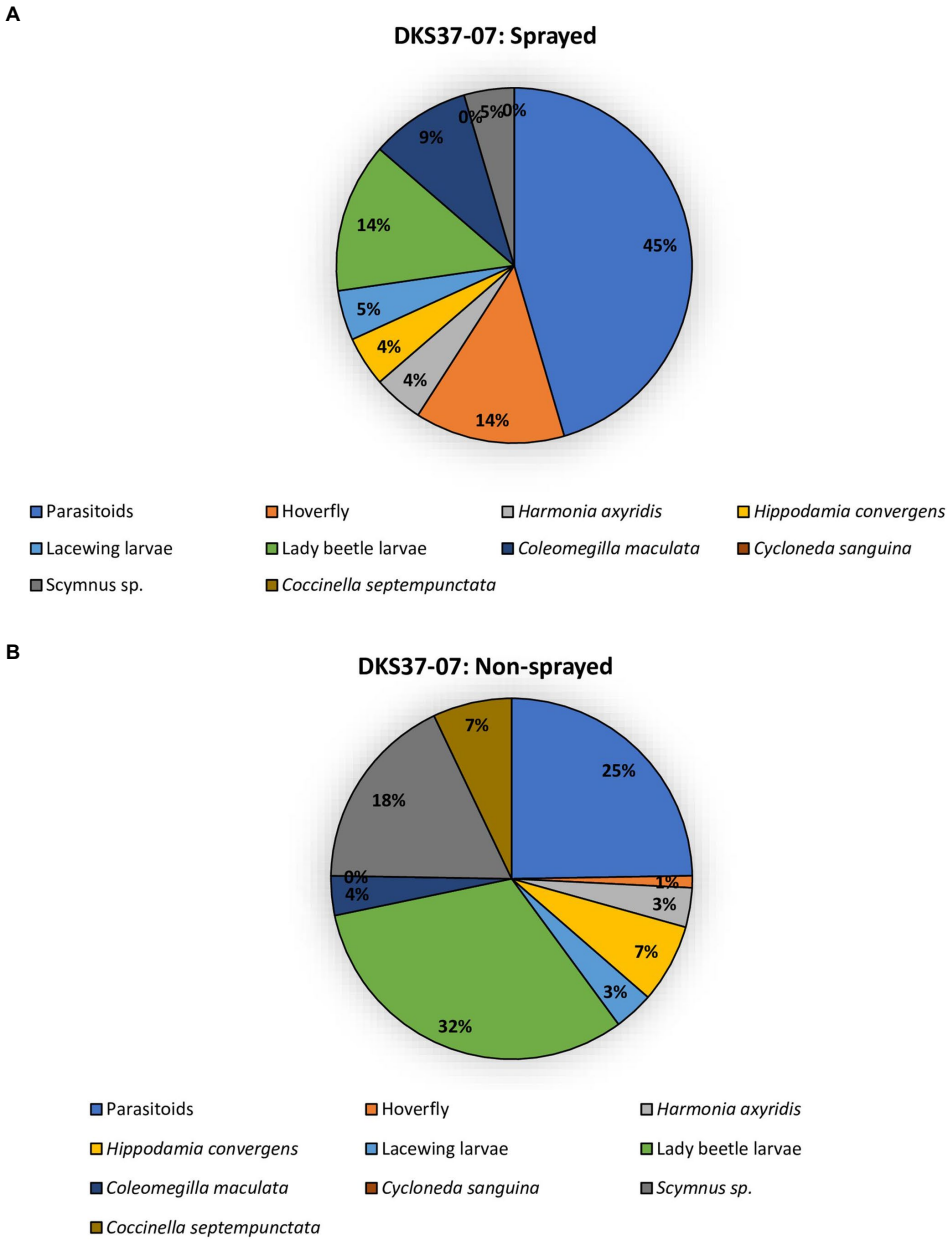
Percentage of natural enemies observed on resistant sorghum cultivar (DKS37-07) that was flupyradifurone sprayed **(A)** or non-sprayed **(B)** using the pooled data from all locations.

**Figure 6:**
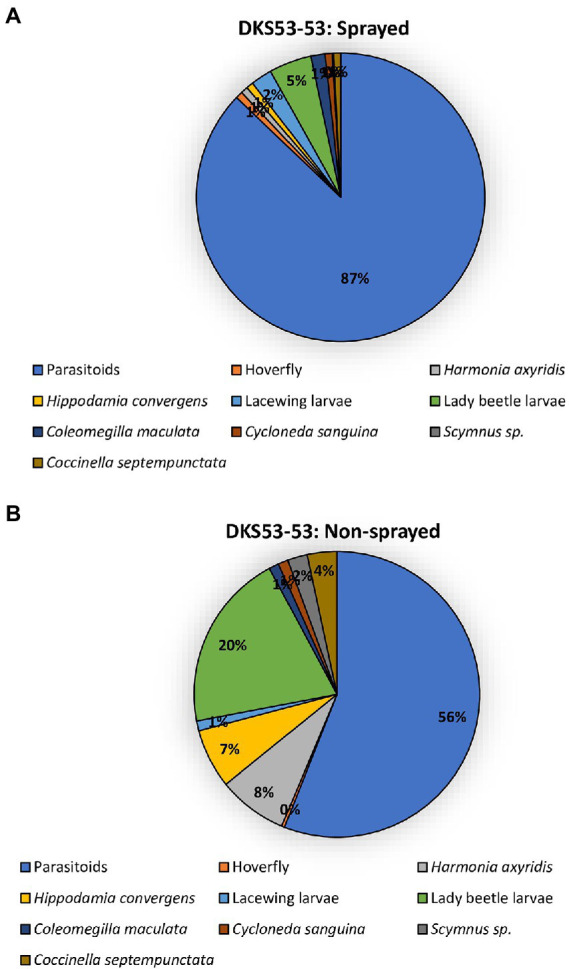
Percentage of natural enemies observed on susceptible sorghum cultivar (DKS53-53) that was flupyradifurone sprayed **(A)** or non-sprayed **(B)** using the pooled data from all locations.

The two sorghum cultivars (DKS37-07 and DKS53-53) supported an array of different predator and parasitoid life stages (larvae and adults) in both the sprayed and non-sprayed treatments (Figures 5, 6). Seven species of adult and larvae of lady beetles [*Coccinella septempunctata* (L.), *Hippodamia convergens* (Guérin-Méneville), *Hippodamia sinuate* (Mulsant), *Coleomegilla maculata* (DeGeer), *Scymnus loewii* (Mulsant), *Cycloneda sanguinea* (L.)] and *Harmonia axyridis* (Pallas; Coleoptera: Coccinellidae), four species of lacewing larvae [*Hemerobius* sp. (Neuroptera: Hemerobiidae), *Ceraeochrysa valida* (Banks), *Chrysopa quadripunctata* Burmeister, and *Chrysoperla plorabunda* (Fitch; Neuroptera: Chrysopidae)], and two parasitoid taxa [*Lysiphlebus testaceipes* (Cresson; Hymenoptera: Braconidae) and *Aphelinus* sp] were recorded for both sprayed and non-sprayed plots of both cultivars (Figures 5, 6). In the foliar insecticide sprayed resistant sorghum hybrid, parasitoids accounted for 45% of the total natural enemy number while lady beetle larvae and *Allograpta obliqua* larvae represented 14% each of the total natural enemies found (Figure 5A). Lady beetle larvae (32%) and parasitoids (25%) were more abundant on the non-sprayed resistant sorghum cultivar (Figure 5B). In the sprayed susceptible sorghum cultivar., parasitoids accounted for 87% of the total natural enemy composition while lady beetle larvae represented 5% of the total natural enemies found (Figure 6A). Lady beetle larvae (56%) and parasitoids (20%) were more abundant on the non-sprayed susceptible sorghum cultivar (Figure 6B).

**Figure 7:**
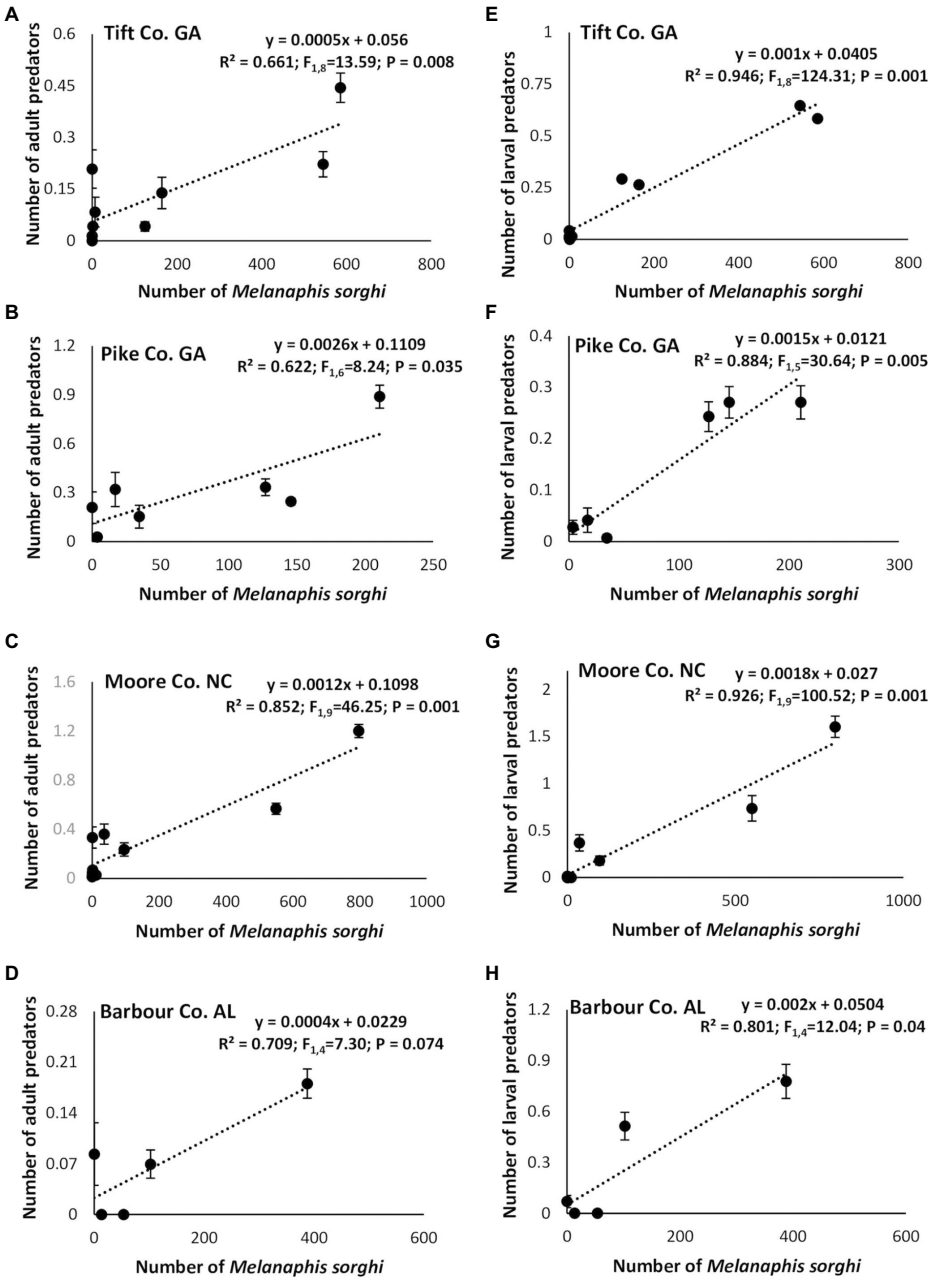
Relationships between mean (±SE) weekly *Melanaphis sorghi* counts and adult **(A–D)** or larval **(E–H)** predators across cultivars, insecticide treatment and locations.

**Figure 8:**
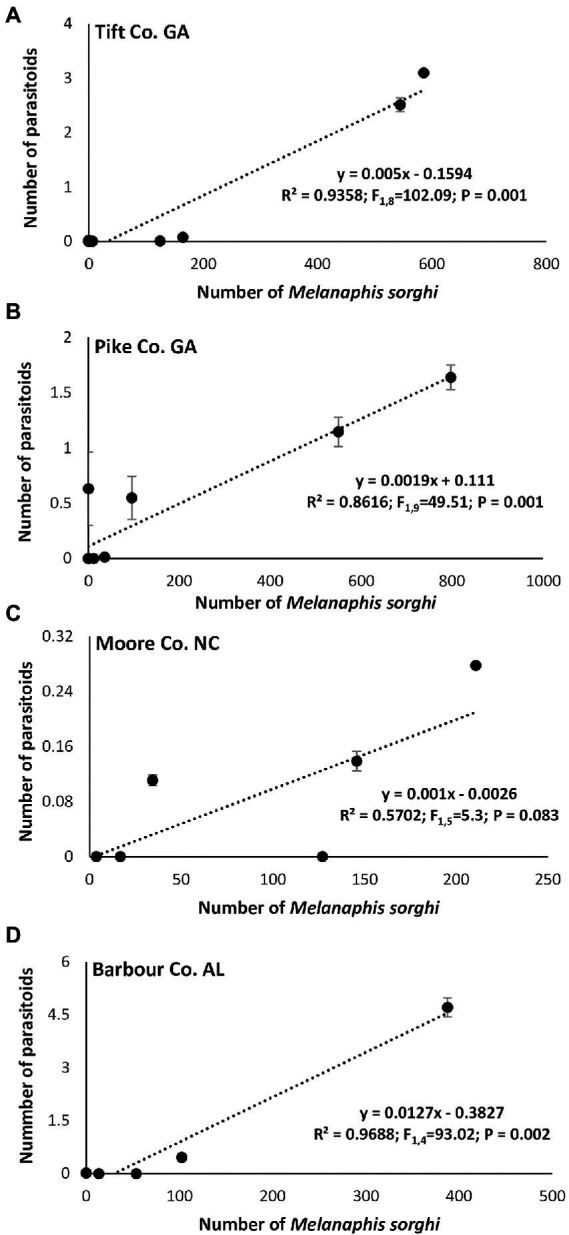
Relationship between mean weekly *Melanaphis sorghi* and parasitoids observed across hybrids, insecticide treatment, and locations.

Linear regression analysis showed a significant positive relationship between the number of *M. sorghi* and number of adult predators in Tift, Pike and Moore Counties (Figures 7A–D) except for Barbour, Co., AL. Similarly, a significant positive relationship between the number of *M. sorghi* and number of larval predators was evident in all four study locations (Figures 7E–H). Finally, there was a significant positive relationship between the number of *M. sorghi* and parasitoids numbers and mummified aphids (Figures 8A–D).

## Discussion

We documented the benefits of combining aphid resistant sorghum cultivar and a single foliar insecticide application of flupyradifurone to suppress *M. sorghi* infestation and reduce yield loss in four locations in southeastern United States. Although within-season plant damage ratings did not vary widely, planting resistant cultivar and foliar application preserved grain yield across locations except under extreme aphid pressure at the Tift Co. study location. Our study also documented a positive association between aphid infestations and the number of natural enemies suggesting that natural enemies do play a role in the integrated pest management of *M. sorghi*. This finding is important because it shows that even a highly efficacious insecticide application may not preserve yield; an integrated approach is necessary.

Identification of environmental factors that drive infestation intensity on a spatio-temporal scale across the invasive range of the pest is key to advancing our understanding of the population ecology of this invasive pest and such studies should be the focus of future research. The mean CID were significantly lower in the insecticide sprayed plots, and on the resistant cultivar (DK37-07) across all locations except Barbour Co., AL where relatively light aphid pressure was observed. The suppression of *M. sorghi* population in this study confirms the reliability of the use of host pant resistance and flupyradifurone application to manage *M. sorghi* across a wide geographic area in the invasive range of the pest in the United States (Szczepaniec, 2018; Lahiri et al., 2021). Tift Co., GA and Moore Co., NC had higher infestations compared to other locations. Changes in weather conditions such as temperature and frequency of rainfall events as well as the presence or absence of natural enemies may influence the severity of *M. sorghi* infestation across spatial scales (Szczepaniec, 2018; Seiter et al., 2019; Souza and Davis, 2020; Wilson et al., 2020). As has been demonstrated by a previous study (Lahiri et al., 2021), differences in CID between sorghum cultivars were most evident when CID was very high compared to locations where CID was low. In southeastern USA grain sorghum, *M. sorghi* infestation intensity often varies among locations and years (Haar et al., 2019; Lahiri et al., 2021).

Across study locations, host plant resistance and the application of flupyradifurone did not significantly influence plant injury except in Tift Co. where the resistant cultivar (DKS37-07) suffered considerably less damage compared to the susceptible cultivar (DKS53-53). Although plant damage ratings did not vary widely in the study, there was generally a positive association between aphids counts and observed plant damage suggesting that increasing aphid numbers resulted in corresponding increase in plant damage.

The lack of significant differences between grain sorghum cultivars and between insecticide treatments, in the overall grain yield (when data from all locations were pooled) shows the importance of location variation in these kinds of experiments (e.g., Lahiri et al., 2021), and further buttress the need for an areawide approach in integrated pest management in sorghum. However, grain sorghum data from individual locations differed either according to sorghum cultivar or insecticide application. Preserved grain yield in plots treated with flupyradifurone application across all locations (except Tift Co.) confirm the findings of previous authors who worked on *M. sorghi* in southeastern United States (e.g., Haar et al., 2019; Lahiri et al., 2021; Pekarcik and Jacobson, 2021). Yields were significantly greater in Moore Co., NC compared to the other study sites. We reason that this difference may be due to a multiplicity of factors including but not limited to rainfall, soil type, weather conditions and other environmental conditions that could influence *M. sorghi* infestation intensity. Host plant resistance had a positive effect on grain yield in Tift and Moore Counties; in Moore Co., no foliar application was required to achieve greater than 500 kg/ha yield in the resistant cultivar (DKS37-07). Yield loss is the cumulative effect of all stresses during the growing year. Aphids represent a major source of stress and can explain some yield results, but other factors such as plant disease, water stress and even bird damage prior to harvest could have suppressed yield potential. In Tift Co., complete yield loss was recorded in experimental plots planted with the susceptible grain sorghum cultivar (DKS53-53) irrespective of insecticide treatment. Studies reporting 100% yield loss in susceptible grain sorghum cultivars grown under intense pressure are not uncommon in the literature (see Brewer et al., 2017; Lahiri et al., 2021).

In addition to using host plant resistance and foliar insecticide application as an effective management tool in *M. sorghi* infestation as described in this current study, we also found that the two sorghum cultivars (DKS37-07 and DKS53-53) supported an array of different life stages of natural enemies (predators and parasitoids) for both the sprayed and non-sprayed treatments. Studies suggest that natural enemies maybe utilized in the *M. sorghi* and grain sorghum system to reduce pest damage and yield loss (Szczepaniec, 2018; Lahiri et al., 2021). Our findings suggest that predators may be more abundant in resistant sorghum (DKS37-07) compared to the susceptible cultivar (DKS53-53). Predators of *M. sorghi* reported in this current study (*C. septempunctata*, *H. convergens*, *H. sinuate*, *C. maculata*, *S. loewii*, *C. sanguinea*, and *H. axyridis*; *Hemerobius* sp., *C. valida*, *C. quadripunctata*, and *C. plorabunda*; *A. obliqua*, *P. clavatus*, and *E. americanus*) have been reported from the invasive range of *M. sorghi* in southeastern United States (Szczepaniec, 2018; Lytle and Huseth, 2021). For both resistant and susceptible cultivars, parasitoids (*L. testaceipes* and *Aphelinus* sp.) were more abundant in the sprayed treatments suggesting that foliar insecticide applications may not have any serious negative effects on the populations of the parasitoids encountered in this study. We hypothesized that the changes in natural enemy populations between sprayed and unsprayed could be a simple reflection of fewer aphids resulting in fewer natural enemies. Differences in predators and parasitoids species abundance or composition are not uncommon because factors including but not limited to weather conditions and prey or host availability can cause their population to spatio-temporally vary (Varenhorst and O’Neal, 2012; Whalen et al., 2016; Lytle and Huseth, 2021).

The strong and significant positive relationship between the natural enemies (larval predators, adults predators and parasitoids numbers) and *M. sorghi* infestation suggests that flupyradifurone application may not have significant negative effects on natural enemy populations. Lytle and Huseth (2021), who studied the impact of insecticide sprays on *M. sorghi* and natural enemy populations in grain sorghum system in North Carolina, also reported that foliar insecticidal treatments did not negatively impact natural enemy populations. The authors showed that by 22 and 29 days after spraying, there were no differences in natural enemy abundance in any treatments including the untreated control.

These findings suggest that the combination of host plant resistance and foliar insecticide application and the presence of natural enemies significantly suppressed *M. sorghi* population and in parts increased yield in grain sorghum. The integration of natural enemies with other conventional control methods in the management of *M. sorghi* comprise an effective integrated pest management strategy against this invasive pest. Our results provide some new insights into the role of natural enemies and other conventional control methods that can enable more informed decisions for growers that are concerned with the balance between insecticide application and biological control for lasting and sustained pest suppression in the *M. sorghi* and grain sorghum system. Given the importance of sorghum and the expansion of sorghum planted areas, in the United States [United States Department of National Agricultural Statistics Services (USDA-NASS), 2017], studies that integrate planting dates with the use of natural enemies and other conventional control approaches could further identify or refine strategies that limit this pest.

## Data availability statement

The original contributions presented in the study are included in the article/supplementary material, further inquiries can be directed to the corresponding author.

## Author contributions

SL, XN, and MT conceptualized the study. MT, SL, XN, DB, AJ, FR-J, SP, and AH collected data and critically reviewed and amended the manuscript. OU and SL performed data analyses. OU wrote the manuscript. All authors contributed to the article and approved the submitted version.

## Funding

This research was supported by a None-Assistance Cooperative Agreement (NACA) 58-6048-6-021 from an areawide pest management project entitled “Area-Wide Pest Management of the Invasive Sugarcane Aphid in Grain Sorghum,” which was awarded to the ARS Research Project 6648-21220-018-00D entitled “Genetic Improvement of Maize and Sorghum for Resistance to Biotic and Abiotic Stresses” and the Alabama Agricultural Experiment Station.

## Conflict of interest

The authors declare that the research was conducted in the absence of any commercial or financial relationships that could be construed as a potential conflict of interest.

## Publisher’s note

All claims expressed in this article are solely those of the authors and do not necessarily represent those of their affiliated organizations, or those of the publisher, the editors and the reviewers. Any product that may be evaluated in this article, or claim that may be made by its manufacturer, is not guaranteed or endorsed by the publisher.
